# Population Health Management in Diabetes Care: Combining Clinical Audit, Risk Stratification, and Multidisciplinary Virtual Clinics in a Community Setting to Improve Diabetes Care in a Geographically Defined Population. An Integrated Diabetes Care Pilot in the North East Locality, Oxfordshire, UK

**DOI:** 10.5334/ijic.5177

**Published:** 2020-11-02

**Authors:** O. Kozlowska, S. Attwood, A. Lumb, G. D. Tan, R. Rea

**Affiliations:** 1 Oxford Brookes University, Headington Campus, Oxford, UK; 2 Bicester Health Centre (retired), UK; 3 Oxfordshire Clinical Commissioning Group (retired), UK; 4 Oxford Centre for Diabetes, Endocrinology & Metabolism, Oxford University Hospitals NHS Foundation Trust, Oxford, UK; 5 NIHR Oxford Biomedical Research Centre, Oxford, UK

**Keywords:** population health, diabetes care, integrated care, service redesign, health disparities

## Abstract

**Background:**

Disparities in diabetes care are prevalent, with significant inequalities observed in access to, and outcomes of, healthcare. A population health approach offers a solution to improve the quality of care for all with systematic ways of assessing whole population requirements and treating and monitoring sub-groups in need of additional attention.

**Description of the care practice:**

Collaborative working between primary, secondary and community care was introduced in seven primary care practices in one locality in England, UK, caring for 3560 patients with diabetes and sharing the same community and secondary specialist diabetes care providers. Three elements of the intervention included 1) clinical audit, 2) risk stratification, and 3) the multi-disciplinary virtual clinics in the community.

**Methods:**

This paper evaluates the acceptability, feasibility and short-term impact on primary care of implementing a population approach intervention using direct observations of the clinics and surveys of participating clinicians.

**Results and discussion:**

Eighteen virtual clinics across seven teams took place over six months between March and July 2017 with organisation, resources, policies, education and approximately 150 individuals discussed. The feedback from primary care was positive with growing knowledge and confidence managing people with complex diabetes in primary care.

**Conclusion:**

Taking a population health approach helped to identify groups of people in need of additional diabetes care and deliver a collaborative health intervention across traditional organisational boundaries.

## Background: health disparities in diabetes care and population health approach

Diabetes is a serious condition that can result in significant morbidity and mortality [1,2,3]. The clinical course and outcome of diabetes depends on how early diabetes is diagnosed and how well the condition is managed. With the growing prevalence of diabetes, health services worldwide find it challenging to provide systematic, ongoing and skilled diabetes care [1]. The World Health Organization recommends sharing the burden of diabetes care between primary and specialist services using standard criteria for referral from primary to secondary or tertiary care [1]. Experience from countries with a long history of referral systems, such as the UK, suggests that a referral process requires specific collaboration between primary and specialist services to work well [4].

Diabetes care in the UK compares reasonably well in comparison with other European nations and beyond [5]. However, focusing on the relatively good outcomes overall can lead to a false conclusion that good quality care is available to everybody. In fact, data from England and Wales show huge geographic variations in care of people with type 1 (T1D) and type 2 (T2D) diabetes [2]. Analysis of these geographical variations highlights the vital role of organisational factors; in particular, the unaddressed split between primary and specialist care has been associated with suboptimal care due to duplication, gaps in provision, delays and fragmentation [2,3,4].

This paper discusses a project designed to address these specific issues. Firstly, it reflects on the split between primary and specialist diabetes care and the impact this has on the quality of diabetes care in populations. Secondly, it proposes a population health approach as a way of improving organisation of diabetes care through specific interventions integrating primary and specialist care. Thirdly, it evaluates the acceptability, feasibility and short-term impact on primary care of implementing a population approach intervention.

### Primary and specialist diabetes care – a split to be mended

In England, care of people with diabetes has been long divided between primary and specialist care. Finding a balance between routine management and specialist input has been an issue since the 1960, up until which time all diabetes care was provided by the specialist services. The growing number of people with T2D prompted the relocation of those suitable for routine management from hospital to primary care [5,6,7]. The discussions about which people should be seen where and when have been continuing ever since [8,9,10] with one of the issues being insufficient supply of consultants in endocrinology [11] and specialist diabetes nurses [12,13].

The challenge of developing an optimal model for people with diabetes has been ongoing. In response to the growing prevalence of diabetes and moving care closer to home to support continuity of care, primary care has been tasked with providing both routine and more complex diabetes care. There is clearly a risk of adverse outcomes for people with diabetes if transfer of responsibility to general practices happens without adequate support [14,15,16] and so it is important to develop an approach for effective and efficient joint working between primary care and specialists to manage the population of people with diabetes under their care. Such an approach widens the population of people with diabetes with access to specialist diabetes support through changes in organisation rather than personnel. Some examples of redefining integrated diabetes care across the UK come from Wolverhampton, Derby, North West London, and Portsmouth, which each propose different models of sharing diabetes care between primary, secondary and community service providers [17,18,19]. With each model came a slightly different understanding of who provides care for which group of people, when and where. The optimal model of integration has yet to be described, and in particular no model thus far provides sufficient methodological detail and evaluation to determine which aspects of integration are essential and which aspects are locally determined. This gap in the literature was identified by the Royal College of Physicians who have developed this as a key piece of transformation work within the Future Hospital Programme [20,21].

### Population Health Approach

A population health approach has the potential to improve the quality of care of individuals by introducing solutions targeting groups and sub-groups at risk of developing complications from diabetes [22,23,24,25]. This approach is a whole system effort to systematically identify, reach and improve care of all individual patients from groups identified as being at risk of poor outcomes. The steps in the process involve measuring health status of a defined group of people and distribution of health outcomes within the group, identifying determinants of health, designing and implementing interventions, and measuring their effectiveness. The methods used in this project included the use of clinical audits, risk stratification, and targeted interventions to provide individuals from the at risk subgroups appropriate and quality care in the right settings, and the use of data to determine changes in outcomes [22].

#### Using clinical audit to improve the quality of healthcare

A good indication of general health disparities can be provided by clinical audits which systematically review the structure, processes and outcomes of care against explicit criteria [26]. There are a number of conditions for an audit to be an effective tool; it needs to exist within a learning and not a blaming culture [27] and be followed with corrective action and supported with resources to deliver change [26,27,28,29].

#### Screening population to identify and monitor individuals at risk of developing complications from diabetes

While a clinical audit indicates the effectiveness of diabetes healthcare on a national, regional, or service level, it does not enable context informed patient level interventions. The latter is provided by a risk stratification process, which identifies those who do not have their diabetes care processes (key diabetes monitoring measurements) done or treatment targets (nationally defined targets for some of these key measurements) achieved and classify them as being at high, medium or low risk of poor outcomes. [30,31,32].

#### Joint working between primary care and specialists – joint clinics to address the audits, risk stratification and clinical decisions

It has been increasingly recognised that a good diabetes care pathway addresses the needs of the local service and is underpinned by a multidisciplinary team working between generalists and specialists, across professional groups, and with specialists reaching into the community [21]. The *multidisciplinary virtual clinics in the community* are one of the options for joint working. The model is for primary care staff to be supported by specialists (diabetes specialist nurses, diabetologists, psychiatrists) through virtual clinics to manage the care of patients with T1D who do not attend the outpatient clinics and of patients with T2D who do not achieve expected treatment outcomes when receiving usual care. ‘Virtual clinic’ refers to face-to-face case conferences between health professionals from primary care and specialists to discuss an individual’s care without their being present [33] as opposed to ‘telemedicine care’ [34,35], or ‘internet-based interventions’ [36].

In diabetes research, joint working is underrepresented [13,37]. The varying specifications for the clinics, the complex interventions poorly described [44,45], and a lack of trials with a few exceptions [33,42,43] make comparing the different models difficult. Although based on the limited evidence, virtual clinics seem feasible, show a positive impact on care processes, and are associated with improved outcomes of care [33,38,39,40,41,43,46,47,48,49,50,51,52,53,54].

## Description of the care practice

### The population health approach to diabetes care in the North East Locality, Oxfordshire

#### The integrated diabetes care project

The project started in 2014 with the aim of improving diabetes care by developing a fully integrated diabetes service across primary and specialist care in Oxfordshire. The objectives were to find a sustainable solution to the growing demand for diabetes care, provide more person-centred care, improve the outcomes of people with diabetes, and reduce the variation in quality and outcomes of diabetes care across the region while at the same time improving its efficiency. The population health approach was chosen as the ideal way of addressing the unmet needs of the studied population.

In the studied diabetes service, the main route to specialist advice was via the referral system with patients being moved to secondary or community care and discharged back to primary care for routine management. The recognition of the need for bringing diabetes expertise into primary care has been ongoing with previous interventions including specialist outpatient clinics in primary care (specialists seeing some patients with T1D in a primary care setting instead of secondary care) and primary care health care professional (HCP) education delivered by the local specialists. This was helping some patients but not all. A move towards more inclusive diabetes care to reach those who were not benefiting from the referral system was needed. The population health approach offered tools to help identify those who were slipping through the net.

#### Development and piloting of the interventions

One locality, a locality being a local group of practices led by a GP, piloted the population health approach and the interventions: 1) the diabetes audit (which became the local diabetes dashboard), 2) risk stratification – the screening programme to identify patients at risk of developing complications from diabetes, 3) the virtual clinics to plan action in response to issues identified using the audit data and patients’ lists. In the North East Locality, in the time of testing the intervention (data collected by the National Diabetes Audit between 1st Jan 2016 and 31st March 2017), there were 365 people with Type 1 diabetes (2,810 across Oxfordshire) and 3,195 people with Type 2 and other types of diabetes (26,095 across Oxfordshire) [55]. The outcomes of the National Diabetes Audit varied for seven practices involved with some practices achieving the expected or above expected care processes and outcomes with others performing below the national average. The initial model for virtual community diabetes clinics was developed through consultation with primary care surgeries from early 2016 and refined in time for the pilot which started in March 2017.

#### The guiding principles in developing the intervention

The programme adopted the principles of quality improvement in diabetes care aiming to make care a) safe (avoidance of unintended or unexpected harm to people during the provision of care, in this case due to unmet needs of people with diabetes living in community), b) effective (bringing diabetes expertise to primary care), c) patient-centred, d) timely, e) efficient, and f) equitable (available to everybody) [56,57].

The programme was a) theory driven (population health approach and three interventions hypothetically linked with expected changes in the original situation), b) process oriented (focus on how change happens), c) participatory (diabetes practitioners were developing, adapting and interpreting the outcomes of the interventions), d) multidisciplinary and multi-method (exploratory methods beyond experimental research), and e) meticulously detailed (detailed descriptions of context and processes) [58].

#### The development stage

The process of developing the virtual clinics was guided by the principles of co-design in service improvement and continual improvement process. The specialists in diabetes began regular engagement with one surgery in early 2016. Initially the meetings were case discussions with cases identified by primary care healthcare professionals, this was later complemented with the discussions of cases identified in systematic searches of the patients’ list. The systematic searches were refined within a few months. The clinics also included discussions of the surgery’s performance in the National Diabetes Audit Core Audit (NDA). Simultaneously, the interested surgeries across the locality were visited to build engagement and collect their feedback on proposed interventions.

This was followed by a survey (August 2016) seeking comments on the individual practices participation in the NDA, factors contributing to the results achieved, and the best way forward; this information was important to understand the primary care perceptions of barriers and facilitators to good quality diabetes care and the contextual factors impacting on the outcomes. Ten participants (eight GPs and two practice managers) completed the survey representing all seven practices in the locality. All participants supported the idea of closer working between generalists and specialists with an easy access to specialist advice and more presence of specialists in the community. The findings from the survey were used to prepare an action plan informed with respondents’ views to feed into the strategy for integrated diabetes care in the pilot locality. The practice managers were approached to schedule the clinics.

### Content and scope of the intervention

#### Assessing population – the National Diabetes Audit and the local diabetes dashboard

The use of the National Diabetes Audit was intended as an indicator of the quality of diabetes care in the pilot locality and the individual practices and to help target resources whether at individual practice level (access to practice nurses with expertise) or across locality (access to conveniently timed patient education). The shortcomings of using the audit data were identified by the primary care staff, namely the lack of local indicators of quality of care and a delay in reporting outcomes. This feedback triggered development of a monthly diabetes dashboard which included local indicators of quality of care as well as national indicators. This enabled almost real-time population data monitoring. The suggestion from primary care was to discuss the results in the context of the practice resources and its population. The shortcomings are the same as with any clinical audit – the data depends on the quality of coding and transparency of the analysis and these were discussed at the meetings to build trust and mutual understanding of the audit.

#### Screening for patients at risk of developing complications from diabetes

Patient lists were screened using criteria agreed by the clinical team to identify adult patients at risk of developing complications from diabetes:

- patients with HbA1c greater than 9% (75 mmol/mol)
- patients with HbA1c lower than 6.5% (48 mmol/mol) on insulin or sulphonylurea
- patients with diabetes and eGFR <30 ml/min

The lists of patients meeting the above criteria were prepared in readiness for the virtual clinics.

#### The multi-disciplinary virtual clinics in the community

The clinics were organised around three main items on the agenda,

- the results of the audit,
- the care of patients identified in the predefined searches (risk stratification), and
- the care of other complex patients as requested by the primary healthcare professionals.

The integral part of joint working between the clinics was case management with the discussions of the patients’ care and decisions made at the virtual clinics, followed with a review of a care plan with a patient, and then with a follow-up at the virtual clinic. The GPs and practice nurses had access to the consultant led email advice line and telephone line between the virtual clinics (Table 1).

**Table 1:** Model of intervention.

Input	Objective	Output

audit/diabetes dashboard	-to identify areas of unmet need in the individual surgeries	-a list of the individual surgery’s outcomes against the local and national average
screening	-to identify patients at risk of developing complications from diabetes	-a list of patients referred to the virtual clinic
virtual clinic	-to discuss diabetes outcomes of the practice (audit/diabetes dashboard)	-action points
	-to discuss care of individual patients identified during screening	-reviewed treatment plans
	-to discuss care of any other patients in need of an urgent review as identified by the primary care health care professionals	-reviewed treatment plans
	-to disseminate information about patient education	-primary care knowledge of available patient education
	-to educate about diabetes treatment and management and highlight local pathways	

## Methods of evaluation

### Evaluating acceptability, feasibility and early impact of the interventions

The aim of this work was to validate the population health approach and the interventions proposed. A mixed-methods evaluation, including observations of the clinics and survey with those involved in the delivery of the virtual clinics, was conducted with the aim of assessing acceptability and feasibility of interventions, and the initial impact of the intervention on the primary diabetes care. Acceptability was defined as the willingness of primary care healthcare professionals and practice managers to engage with all three interventions. Feasibility was defined as the ability and capacity to deliver them. The impact was defined as any changes in knowledge and confidence in managing diabetes care in primary care, changes in patterns in communicating with and referring to specialist diabetes services, and any changes introduced to diabetes care in primary care due to the interactions with the specialists.

### Data collection

#### Observations of the virtual clinics

The clinics were observed by a researcher with doctoral training in qualitative methodologies assessing the delivery of interventions to ensure they were provided in accordance with the protocol and delivered consistently, if the participants adhered to the protocol, what modifications were made, and explore participants’ views of and satisfaction with the intervention.

In addition, information about each case discussion was recorded to identify how many patients were discussed per clinic, what aspects of their care were discussed, who contributed, what aspects of care were changed, how the decision was made, and if the tasks were assigned. After the first clinic, the following was recorded: were the actions communicated with all interested, were the actions completed, what were the barriers to completing the actions.

#### Survey with healthcare professionals

Recruitment: All practice managers were approached with a request to complete the survey and send the invitation to the GPs and practice nurses in their practices who participated in the clinics. The clinical lead for specialist diabetes nurses was directly invited to participate and asked to invite specialist diabetes nurses participating in the pilot.

Method: A survey consisted of a mix of Likert scales to determine the degree of perceived change to the GPs and primary care nurses’ knowledge about diabetes management, confidence in managing patients with diabetes, and behaviours in clinical practice, and open-ended questions to collect further details of experiences and impact of participating in the clinics. Separate surveys with open-ended questions were conducted with the practice managers, specialist diabetes nurses, and consultants in diabetes to collect their feedback on the changes to the management of diabetes care in primary care and processes involved in the clinics. The survey was conducted over three weeks seven months after the pilot concluded (January 2018).

## Results

### Observations

Eighteen integrated virtual community diabetes clinics took place between March and July 2017 across seven practices in the North East Locality. Each practice held at least two clinics with one practice holding four. Each clinic was attended by at least one GP, one practice nurse, one specialist diabetes nurse, and one consultant in diabetes. Five clinics were attended by one or more mental health specialists.

Approximately 150 patients were discussed, on average eight patients per 60 minute clinic, with the number depending on the complexity of cases, the level of preparation of the case by primary care, the availability of information from the IT system and speed of operating it, the experience of working together and any previous discussions of patients. The majority of patients had type 2 diabetes, 23 had type 1 diabetes, and 3 had a type yet to be confirmed.

The interventions changing the course of diabetes care included:

- change of medication
- introducing new medication
- introducing new intervention, e.g. diet, lifestyle change, referral for consideration of bariatric surgery
- change of medication dose
- referring to diabetes specialist teams in secondary or community care
- referring to allied specialities including dieticians and mental health specialists
- referring to patient structured education
- agreeing on delivering specialist care in primary care
- agreeing on complex care pathway with primary care delivering initial intervention, and if not effective, scaling up to the specialist service
- reassuring primary care healthcare professionals about appropriateness of interventions implemented

The feedback from primary care voiced during the virtual clinics was positive with the following noted:

- the clinics were seen as educational sessions with useful guidelines and advice
- knowledge gained at the clinics was applicable and transferable to patient cases not discussed at the virtual clinics
- the clinics increased understanding of diabetes services available and the referral system

### Survey

#### Participation

Thirteen participants responded to the survey including 5 GPs (out of at least 7), 2 primary care nurses (out of at least 7), 4 practice managers (out of 7) and 2 diabetes specialist nurses (out of 3). Five out of 7 participating practices were represented.

#### Perceived changes in knowledge and confidence in managing diabetes among primary care healthcare professionals

The self-reported knowledge of diabetes management and referral system gains were reported by all primary care healthcare professionals (GPs and primary care nurses) in all or some aspects of diabetes care following the clinics. The self-reported change in confidence in managing diabetes varied and was different depending on the type of diabetes. Table 2 provides an overview of how many GPs and primary care nurses observed changes, or its lack, in their knowledge and confidence.

**Table 2:** Perceived changes in knowledge and confidence in managing diabetes among primary care healthcare professionals (GPs and primary care nurses) following the pilot.

	Stayed insufficient	Stayed sufficient	Increased

**Confidence in managing patients with diabetes**			
confidence of managing patients with type 1 diabetes	3	2	2
confidence of managing patients with type 2 diabetes		2	5
**Knowledge of management of diabetes**			
knowledge of administering diabetes medication		2	5
knowledge of non-pharmacological diabetes treatment options, e.g. lifestyle changes, bariatric surgery		6	1
knowledge of local diabetes guidelines		4	3
knowledge of national diabetes guidelines		3	4
knowledge of psychological needs of people with diabetes	1	3	3
knowledge of mental health problems linked to diabetes	1	3	3
**Knowledge of the referral system**			
knowledge of the referral system to specialist diabetes nurses		4	3
knowledge of the referral system to diabetes specialists		2	5
knowledge of the referral system to mental health services	1	3	3
knowledge of the referral system to patient structured education		3	4
knowledge of the referral system to diabetes specialist dietitian	2	4	1

#### Self-reported change in primary care contacts with specialists

All primary care healthcare professionals (GPs and primary care nurses) reported an increase in their contacts with the diabetes consultants and majority with the specialist diabetes nurses (DSNs). The impact was noticed in terms of the number of referrals to different specialist services and Table 3 provides information on how many respondents observed a change, or its lack, in their referral pattern.

**Table 3:** Self-reported change in primary care contacts with specialists following the pilot.

	Decreased	Stayed insufficient	Stayed sufficient	Increased

contacts with diabetes specialists				7
contacts with specialist diabetes nurses			2	5
referrals to diabetes specialists			4	3
referrals to specialist diabetes nurses	2		3	2
referrals to mental health services		1	3	3
referrals to diabetes specialist dietitian		3	3	1
referrals to patient structured education			2	5

It is unclear if the increase or decrease of referrals is a positive or negative change. The increased awareness of diabetes issues and number of patients identified as in need of further attention increased the referral rates as reported by one GP, while education provided at the clinics reduced referrals to the DSNs as observed by another GP. The DSNs confirmed that the frequency of seeking their advice increased as well as requests for face to face assessment; though the increase was seen as a positive change, there were concerns that the referrals were not always appropriate.

#### Changes to the management of diabetes in the practices

Across the practices, the primary care staff (GPs, primary care nurses, practice managers) reported changes on the practice level that have been introduced following the clinics:

- adopting the Year of Care approach promoted at the clinics
- continuing with risk stratification and searching the patient lists for patients at risk
- focusing more on blood pressure in patients (specific interventions included: educating healthcare assistant on BP targets, home BP monitoring, trying a 3/6 months recall date)
- setting up a new alert for those who did not have microalbuminuria checked in last year
- checking more often whether a low HbA1c could mean regular hypos
- picking up the issues of unrecognised hypoglycaemia in patients on insulin and gliclazide
- bringing people with high HbS1c with diabetes in more frequently
- altering insulin doses
- actively tracking and approaching disengaged patients

#### Satisfaction with the intervention

There was a shared feeling that the pilot should be extended with further work to refine it. The feedback coming from all participants was positive and the face-to-face format of the meetings was appreciated by both primary care HCPs and DSNs. In overall, all primary care HCPs reported following the decisions made at the clinics but some experienced problems in implementing them due to poor patients’ engagement or internal administration issues. As the practice managers emphasised, the clinics increased focus on diabetes in the practices. Together with other training in diabetes provided by the consultants and DSNs the interventions complemented each other.

### Main outputs

The key outputs of the pilot, identified from the observations, voiced feedback, and survey, included changes in the processes related to management of diabetes in primary care; identification of the gaps in knowledge of diabetes and its management among GPs and primary care nurses; new ways of working between GPs, primary care nurses and diabetes specialists; and raising awareness of diabetes research. In particular, in each of the areas, the outputs included:

- management of diabetes in primary care
  - plans to continue with the virtual clinics beyond the pilot; all practices expressed a willingness to do so
  - identifying groups of patients in need of intervention but previously not perceived as such by primary care
  - providing primary care practices with a tool (searches) to systematically screen their population and identify patients in need for intervention
  - producing a protocol for the virtual clinics to be used across Oxfordshire
  - designing a new format of the outpatient letter to primary care including relevant information (e.g. care processes delivered) presented in a systematic way
  - improving recording of information in primary care for the National Diabetes Audit by exchanging information during the virtual clinics (patient structured education)
- education of primary care healthcare professionals
  - challenging diabetes treatments and management not aligned with national or local guidelines
  - raising awareness of a range of diabetes treatments and interventions available within primary care
  - changing a narrative about people with high HbA1c – shifting of blame in poorly controlled diabetes from patients to complexity of condition with its physical and mental health aspects, unresponsiveness of the health services and gaps in the service
- developing a community of well-linked practitioners
  - improving the linkage between services with new referrals being made at the virtual clinics to secondary care, community care, patient structured education, mental health specialists, and community type 1 diabetes clinics
  - planning together location and level of care in consideration of patients’ needs and not primary and secondary care boundaries
- research
  - increasing number of people recruited into clinical trials

## Discussion

### Interpretation of the findings in context of previous research

The pilot confirmed the acceptability and feasibility of the population health approach and interventions in primary care in an environment with a limited previous experience of joint working. Key gaps in knowledge and confidence in managing patients with diabetes and knowledge of referral practices were identified and addressed through the virtual clinics. The clinics provided a space to explore and address the benefits and problems of joint working.

One of the key benefits of the project was the development of a monthly local clinical audit which extracted NDA data from GP surgeries and was able to show improvements in a short period of time. Essential to the success of this audit tool was a learning not blaming atmosphere [27] with the GPs, primary care nurses and specialists discussing the diabetes outcomes of the individual general practices to identify challenges, strengths, contextual factors impacting on the outcomes, and work together towards solutions, and also following the audit with an action plan and ongoing monitoring [26,27]. In a short time, the most recent written national and local guidelines, previously not fully implemented by primary care, were brought to the attention of primary care and implemented [26,28,49].

The screening of their population in search of those at risk of poor outcomes enabled the practices to look at the individual patients as part of bigger groups with shared problems and plan for care sharing primary care and specialist resources [30,31,32]. The virtual clinics, with the complexity of processes and decision making involved, took a considerable amount of time to develop during which time trust was being built, appreciation of each other’s contributions recognised, ways of conducting the meetings refined, collaboration outside of the meetings worked out, and responsibilities allocated [52,53,54].

The study highlights the importance of focusing on the different processes involved in changing healthcare across a number of different organisations, and acknowledging the multicomponent and multifactorial nature of such interventions [44,45,58]. Being theory driven, process oriented, participatory, multidisciplinary, multi-method, and meticulously detailed (as the project progressed) contributed to making the change feel justified and the process transparent. The service was driven by the ambitious aims of improving diabetes care for the whole population and was guided by the above principles. This helped the project to reflect when change was not happening as quickly as anticipated and provide a realistic assessment of what was achievable at a particular time.

### Implications for future practice and research

The interventions tested in the pilot continue to be used in the piloted locality and across the county. The context has changed as the pilot was replaced with a voluntary paid service with practices hosting specialists twice a year. The service has been sustained through the NHS England Diabetes Transformation Funding programme and has been shown to improve not only healthcare professional confidence but also the key care processes delivered to patients. It is currently being refined along the lines of new Primary Care Networks and is now a part of the Locally Commissioned Service for Diabetes within Oxfordshire. Further work is required to refine the processes involved in the virtual clinics including investigating video interactions, a process which has been accelerated by the Covid-19 outbreak, and ongoing review of high risk patients within the GP surgery in between the virtual clinics. A broader impact assessment on the wider health economy including admissions as well as morbidity arising from diabetes is required now that the pilot has been rolled out across the county.

### Limitations

This work would benefit from more complex evaluation of the intervention without relying on self-reports only when assessing changes in knowledge and confidence of healthcare professionals. Also, more insights from the participating HCPs on why some of the interventions did not meet their needs and what further improvements are needed. Unfortunately, the reasons for it not happening (e.g. no increase in knowledge or confidence in managing diabetes) for some were not explored further. It is too early to assess the impact of changes in referral patterns following HCP education of the services available for people with diabetes.

## Conclusions

The implementation of virtual clinics successfully piloted a population health approach in diabetes care, focusing on population screening, risk stratification and assessment (identifying patients at high risk of complications), reviewing patient cases (identifying solutions that are applicable across the population), and improving individual patients’ care as well as at practice and population level. The multidisciplinary virtual clinics in the community enabled the service to

1. discuss the outcomes of audit taking into consideration the characteristics of the population and plan for improvement,
2. proactively identify groups of patients at risk of complications from diabetes, and
3. plan their care together.

Continuing diabetes education in primary care focused on building expertise and skills rather than the dissemination of guidelines. Unnecessary referrals were avoided by encouraging shared decision making and shared responsibility for treatment changes. The intervention had an educational value, improved confidence in primary care in managing diabetes, and improved communication between primary care and specialists. It made the diabetes care for the whole population more cohesive in the piloted area.

This project developed and delivered a geographical based intervention to improve care for people with diabetes starting from a baseline of a traditional model of care delivery. Factors which enabled successful adaptation and delivery of a high quality service included the establishment of working relationships and trust developed from engaging with each practice individually to discuss their specific circumstances, allowing for flexibility in the organisation of virtual clinics, working in primary care settings, appreciating primary care familiarity with patients and specialist subject area expertise, and collecting. All of this was underpinned by ongoing reporting of data and acting on ongoing feedback from primary care.

## Lessons learnt

- Local engagement is essential in the development of the model
- Regular updated trusted data is essential to show improvement and understand what changes are effective
- A culture of learning and support is essential rather than blame and performance management
- Face to face engagement between GPs, primary care nurses, and specialists outside of the hospital and in the community can transform relationships and break down barriers to joint working

## References

[1] World Health Organisation. Global Report on Diabetes Geneva: WHO; 2016 [cited 26.02.2019]. Available from: https://apps.who.int/iris/bitstream/handle/10665/204871/9789241565257_eng.pdf;jsessionid=CCB21E4833142B3B04CAE43BAE4C2E32?sequence=1.

[2] NHS Digital. National Diabetes Audit, 2016–17. Report 1: Care Processes and Treatment Targets, England and Wales. Full report; 2018 [cited 27.02.2019]. Available from: https://files.digital.nhs.uk/pdf/s/k/national_diabetes_audit_2016-17_report_1__care_processes_and_treatment_targets.pdf.

[3] NHS England, Medical Directorate. Action for Diabetes. 2014 [cited 14.03.2019]. Available from: https://www.england.nhs.uk/rightcare/wp-content/uploads/sites/40/2016/08/act-for-diabetes-31-01.pdf.

[4] Diabetes UK. Best practice for commissioning diabetes services: An integrated care framework. 2013 [cited 20.11.2018]. Available from: https://diabetes-resources-production.s3-eu-west-1.amazonaws.com/diabetes-storage/migration/pdf/best-practice-commissioning-diabetes-services-integrated-care-framework-0313.pdf.

[5] Garrofé BC, Björnberg A, Yung Phang A. Euro Diabetes Index. Health Consumer Powerhouse Ltd 2014 Available online at: https://old.healthpowerhouse.com/files/EDI-2014/EDI-2014-report.pdf [cited 17.02.2019].

[6] Malins JM, Stuart JM. Diabetic clinic in a general practice. British Medical Journal, 1971; 4: 161 DOI: 10.1136/bmj.4.5780.161 5113020 PMC1799021

[7] Thorn PA, Russell RG. Diabetic clinics today and tomorrow: mini-clinics in general practice. British Medical Journal, 1973; 2: 534–536. DOI: 10.1136/bmj.2.5865.534 4714471 PMC1589562

[8] Wood J. A review of diabetes care initiatives in primary care settings. Health Trends, 1990; 22(1): 39–43. 10106791

[9] Worswick J, Wayne SC, Bennett R, Fiander M, Mayhew A, Weir MC, Sullivan KJ, Grimshaw JM. Improving quality of care for persons with diabetes: an overview of systematic reviews – what does the evidence tell us? Systematic reviews, 2013; 2: 26 DOI: 10.1186/2046-4053-2-26 23647654 PMC3667096

[10] Wilkes E, Lawton EE. The diabetic, the hospital and primary care. Journal of the Royal College of General Practitioners, 1980; 30: 199–206. 7411509 PMC2159470

[11] Royal College of Physicians Focus on physicians. Census of consultant physicians and higher specialty trainees 2017–18. 2017–18 consultant census data tool. 2018 Available online at: https://www.rcplondon.ac.uk/file/11458/download?token=B66sa0_H [cited 18.03.2019].

[12] Gosden JJ, Wincourt P, Walton C, Nagi D, Turner B, Williams R, Holt RIH. Diabetes specialist nurses and role evolvement: a survey by Diabetes UK and ABCD of specialist diabetes services 2007. Diabetic Medicine, 2007; 26: 560–565. DOI: 10.1111/j.1464-5491.2009.02716.x 19646199

[13] Diabetes UK. Diabetes specialist nursing 2016 workforce survey: A workforce in crisis. London: DUK 2016 Available online at: http://bit.ly/2gOi2EU [cited 21.02.2019].

[14] Price E, Baker R, Krause J, Keen C. Organisation of services for people with cardiovascular disorders in primary care: transfer to primary care or to specialist-generalist multidisciplinary teams? BMC Fam Pract., 2014; 15: 158 DOI: 10.1186/1471-2296-15-158 25245456 PMC4262997

[15] Duran A, Runkle I, Matía P, de Miguel MP, et al. Family physician and endocrinologist coordination as the basis for diabetes care in clinical practice. BMC Endocr Disord., 2008 7 31; 8: 9 DOI: 10.1186/1472-6823-8-9 18671870 PMC2518542

[16] Griffin S. Diabetes care in general practice: meta-analysis of randomised control trials. BMJ, 1998; 317: 390 DOI: 10.1136/bmj.317.7155.390 9694757 PMC28634

[17] Diabetes UK. Improving the delivery of adult diabetes care through integration. Sharing experience and learning. London: DUK 2014 Available online at: https://diabetes-resources-production.s3-eu-west-1.amazonaws.com/diabetes-storage/migration/pdf/Integrated%2520diabetes%2520care%2C%2520Diabetes%2520UK%2520%28PDF%2C%2520648KB%29.pdf [cited 13.09.2019].

[18] Kar P. The Super Six Model: Integrated diabetes care across Portsmouth and south-east Hampshire. Diabetes & Primary Care, 2012; 14(5): 277–283.

[19] Wills T, Chandok R, Delgado R, Robertson L, Dornhorst A. The North West London Diabetes Transformation Programme (NWL-DTP): the journey so far in optimising diabetes care within North West London. Practical Diabetes, 2019; 36(2): 51–56. DOI: 10.1002/pdi.2214

[20] Future Hospital Commission. Future Hospital: caring for medical patients A report from the Future Hospital Commission to the Royal College of Physicians. London: Royal College of Physicians; 2013.

[21] Royal College of Physicians. Delivering the Future Hospital Full Report. London: Royal College of Physicians 2017 Available online at file:///C:/Users/p0085541/Downloads/FHC%20final%20report_0_0%20(3).pdf [cited 17.09.2019].

[22] Golden SH, Maruthur N, Mathioudakis N, Spanakis E, Rubin D, Zilbermit M, Hill-Briggs F. The case for diabetes population health improvement: evidence-based programming for population outcomes in diabetes. Curr Diab Rep, 2017; 17(7): 51 DOI: 10.1007/s11892-017-0875-2 28567711 PMC5553206

[23] Schmittdiel JA, Gopalan A, Adams AS. Population Health Management for Diabetes: Health Care System-Level Approaches for Improving Quality and Addressing Disparities. Curr Diab Rep, 2017; 17(5): 31 DOI: 10.1007/s11892-017-0858-3 28364355 PMC5536329

[24] Kindig DA, Stoddart G. What Is Population Health? American Journal of Public Health, 2003; 93: 366–69. DOI: 10.2105/AJPH.93.3.366 12604476 PMC1447747

[25] Dunn JR, Hayes MV. Towards a lexicon of population health. Can J of Pub Health, 1999; 90 Suppl 1: S7–10. DOI: 10.1007/BF03403570 10686751 PMC6979857

[26] Parsons JA, Yu CH, Baker NA, Mamdani MM, Bhattacharyya O, Zwarstein M, Shah BR. Practice Doesn’t Always Make Perfect: A Qualitative Study Explaining Why a Trial of an Educational Toolkit Did Not Improve Quality of Care. PLoS One, 2016; 11(12): e0167878 DOI: 10.1371/journal.pone.0167878 28030547 PMC5193379

[27] Johnston G, Crombie IK, Davies HTO. Reviewing audit: barriers and facilitators for effective clinical audit. Quality in Health Care, 2000; 9: 23–36. DOI: 10.1136/qhc.9.1.23 10848367 PMC1743496

[28] Giguère A, Legare F, Grimshaw J, Turcott S, Fiander M, Grudniewicz A, et al. Printed educational materials: effects on professional practice and healthcare outcomes. Cochrane Database of Systematic Reviews, 2012; (10): 1–197. DOI: 10.1002/14651858.CD004398.pub3 PMC7197046 23076904

[29] Ivers NM, Tu K, Young J, Francis JJ, Barnsley J, Shah BR, Upshur RE, Moineddin R, Grimshaw JM, Zwarenstein M. Feedback GAP: pragmatic, cluster-randomized trial of goal setting and action plans to increase the effectiveness of audit and feedback interventions in primary care. Implementation science: IS, 2003; 8: 142 DOI: 10.1186/1748-5908-8-142 PMC3878579 24341511

[30] Aronson R, Orzech N, Ye C, Brown RE, Goldenberg R, Brown V. Specialist-Led Diabetes Registries and Prevalence of Poor Glycemic Control in Type 2 Diabetes: The Diabetes Registry Outcomes Project for A1C Reduction (DROP A1C). Diabetes Care, 2016; 39: 1711–1717. DOI: 10.2337/dc15-2666 27515966

[31] Munch L, Arreskov AB, Sperling M, Overgaard D, Knop FK, Vilsbøll T, Røder ME. Risk stratification by endocrinologists of patients with type 2 diabetes in a Danish specialised outpatient clinic: a cross sectional study. BMC Health Services Research, 2016; 16(124). DOI: 10.1186/s12913-016-1365-y PMC4826533 27061722

[32] Clark CM, Snyder JW, Meek RL, Stutz LM, Parkin CG. A systematic approach to risk stratification and intervention within a managed care environment improves diabetes outcomes and patient satisfaction. Diabetes Care, 24(6): 1079–1086. DOI: 10.2337/diacare.24.6.1079 11375374

[33] Basudev N, Crosby-Nwaobi R, Thomas S, et al. A prospective randomized controlled study of a virtual clinic integrating primary and specialist care for patients with type 2 diabetes mellitus. Diabet Med, 2016; 33: 768–76. DOI: 10.1111/dme.12985 27194175

[34] Raymond JK, Berget CL, Driscoll KA, Ketchum K, Cain C, Fred Thomas JF. CoYoT1 Clinic: Innovative Telemedicine Care Model for Young Adults with Type 1 Diabetes. Diabetes Technology and Therapeutics, 2016; 18(6): 385–90. DOI: 10.1089/dia.2015.0425 27196443 PMC5583551

[35] Fatehi F, Martin-Khan M, Smith AC, Russell AW, Gray LC. Patient satisfaction with video tele consultation in a virtual diabetes outreach clinic. Diabetes Technology and Therapeutics, 2015; 17(1): 43–8. DOI: 10.1089/dia.2014.0159 25296189

[36] Powell J, Jennings A, Armstrong N, Dale J. Pilot study of a virtual clinic: satisfaction and usability. Journal of Telemedicine and Telecare, 2009; 15(3): 150–2. DOI: 10.1258/jtt.2009.003014 19364901

[37] Murphy ME, Byrne M, Galvin R, Boland F, Fahey T, Smith SM. Improving risk factor management for patients with poorly controlled type 2 diabetes: a systematic review of healthcare interventions in primary care and community settings. BMJ Open, 2017; 7: e015135 DOI: 10.1136/bmjopen-2016-015135 PMC5724222 28780542

[38] Foy R, Hempel S, Rubenstein L, Suttorp M, Seelig M, Shanman R, Shekelle PG. Meta-analysis: Effect of Interactive Communication Between Collaborating Primary Care Physicians and Specialists. Annals of Internal Medicine, 2010; 152: 247–258. DOI: 10.7326/0003-4819-152-4-201002160-00010 20157139

[39] Russell AW, Donald M, Bor SJ, Zhang J, Burridge L, Ware RS, Begum N, McIntyre HD, Jackson CL. Clinical outcomes of an integrated primary–secondary model of care for individuals with complex type 2 diabetes: a non-inferiority randomised controlled trial. Diabetologia, 2019; 62(1): 41–52. DOI: 10.1007/s00125-018-4740-x 30284015

[40] Ritholz MD, Beverly EA, Abrahamson MJ, Brooks KM, Hultgren BA, Weinger K. Physicians’ perceptions of the type 2 diabetes multi-disciplinary treatment team: a qualitative study. Diabetes Educ, 2011; 37(6): 794–800. DOI: 10.1177/0145721711423320 22002972 PMC3707496

[41] McGill M, Blonde L, Chan JCN, Khunti K, Lavalle FJ, Bailey CJ. The Global Partnership for Effective Diabetes Management The interdisciplinary team in type 2 diabetes management: Challenges and best practice solutions from real-world scenarios. J Clin Transl Endocrinol, 2017; 7: 21–27. DOI: 10.1016/j.jcte.2016.12.001 29067246 PMC5651292

[42] Armstrong N, Baines D, Baker R, Crossman R, Davies M, et al. A cluster randomised controlled trial of the effectiveness and cost-effectiveness of Intermediate Care Clinics for Diabetes (ICCD): Study protocol for a randomised controlled trial. Trials, 2012; 13: 164 DOI: 10.1186/1745-6215-13-164 22971356 PMC3512506

[43] Wilson A, O’Hare JP, Hardy A, Raymond N, Szczepura A, Crossman R, Baines D, Khunti K, Kumar S, Saravanan P. Evaluation of the Clinical and Cost Effectiveness of Intermediate Care Clinics for Diabetes (ICCD): A Multicentre Cluster Randomised Controlled Trial. PLoS One, 2014; 9(4): e93964 DOI: 10.1371/journal.pone.0093964 24736243 PMC3988031

[44] Bongaerts BW, Mussig K, Wens J, Lang C, Schwarz P, Roden M, Rathmann W. Effectiveness of chronic care models for the management of type 2 diabetes mellitus in Europe: a systematic review and meta-analysis. BMJ Open, 2017; 7: e013076 DOI: 10.1136/bmjopen-2016-013076 PMC5372084 28320788

[45] Greenhalgh T. Commentary: Meta-analysis is a blunt and potentially misleading instrument for analysing models of service delivery. BMJ, 1998; 317: 395–396. DOI: 10.1136/bmj.317.7155.390 9729094

[46] Nocon A, Leese B. The role of UK general practitioners with special clinical interests: implications for policy and service delivery. Br J Gen Pract, 2004; 54: 50–56. 15002420 PMC1314780

[47] Sharp PS. Glycaemic outcomes following discharge from an intermediate care diabetes clinic. Practical Diabetes International, 2010; 27: 53–54. DOI: 10.1002/pdi.1443

[48] Halfyard C, McGowan D, Whyte M, Gayle C. Diabetes rapid access clinic: a bridge between primary and secondary care (Diabetes UK poster presentation). Diabetic Medicine, 2010; 27: 137.

[49] Tricco AC, Antony J, Ivers NM, Ashoor HM, Khan PA, Blondal E, Ghassemi M, MacDonald H, Chen MH, Kark Ezer L, Straus SE. Effectiveness of quality improvement strategies for coordination of care to reduce use of health care services: a systematic review and meta-analysis. CMAJ, 2014; 186(15): E568–E578. DOI: 10.1503/cmaj.140289 25225226 PMC4203622

[50] Borgermans L, Godersi G, Van Den Broeke C, Verbeke G, Carbonez A, et al. Interdisciplinary diabetes care teams operating on the interface between primary and specialty care are associated with improved outcomes of care: findings from the Leuven Diabetes Project. BMC Health Services Research, 2009; 9: 179 DOI: 10.1186/1472-6963-9-179 19811624 PMC2762969

[51] Greer R, Boulware E. Reducing CKD risks among vulnerable populations in primary care. Adv Chronic Kidney Dis, 2015; 22(1): 74–80. DOI: 10.1053/j.ackd.2014.06.003 25573516 PMC4291538

[52] Tricco AC, Ivers NM, Grimshaw JM, Moher D, Turner L, et al. Effectiveness of quality improvement strategies on the management of diabetes: a systematic review and meta-analysis. Lancet, 2012; 379: 2252–2261. DOI: 10.1016/S0140-6736(12)60480-2 22683130

[53] Shojania KG, Ranji SR, McDonald KM, Grimshaw JM, Sundaram V, Rushakoff RJ, Owens DK Effects of Quality Improvement Strategies for Type 2 Diabetes on Glycemic Control. A Meta-Regression Analysis. JAMA, 2006; 296(4): 427–440. DOI: 10.1001/jama.296.4.427 16868301

[54] Lamb BW, Sevdalis N, Vincent C, Green JS. Development and Evaluation of a Checklist to Support Decision Making in Cancer Multidisciplinary Team Meetings: MDT-QuIC. Annals of Surgical Oncology, 2011; 19(6): 1759–65. DOI: 10.1245/s10434-011-2187-0 22207050

[55] NHS Digital. National Diabetes Audit, 2016–17. Report 1: Care Processes and Treatment Targets, England and Wales. Full report; 2018 Available online at: https://files.digital.nhs.uk/pdf/s/k/national_diabetes_audit_2016-17_report_1__care_processes_and_treatment_targets.pdf [cited 27.02.2019].

[56] Lu BJ, Danko KJ, Elfassy MD, Welch V, Grimshaw JM, Ivers NM. Do quality improvement initiatives for diabetes care address social inequities? Secondary analysis of a systematic review. BMJ Open, 2018; 8: e018826 DOI: 10.1136/bmjopen-2017-018826 PMC5829812 29444781

[57] Institute of Medicine (US) Committee on Quality of Health Care in America. Crossing the Quality Charm. Washington, DC: National Academic Press, US 2001 Available online at: http://www.nationalacademies.org/hmd/~/media/Files/Report%20Files/2001/Crossing-the-Quality-Chasm/Quality%20Chasm%202001%20%20report%20brief.pdf [cited 17.02.2019].

[58] Greenhalgh T, Robert G, Bate SP, Macfarlane F, Kyriakidou O. Diffusion of Innovations in Health Service Organisations. Oxford: Blackwells; 2005 DOI: 10.1002/9780470987407

